# Comparison of Free Total Amino Acid Compositions and Their Functional Classifications in 13 Wild Edible Mushrooms

**DOI:** 10.3390/molecules22030350

**Published:** 2017-02-24

**Authors:** Liping Sun, Qiuming Liu, Changjun Bao, Jian Fan

**Affiliations:** Yunnan Institute of Food Safety, Kunming University of Science and Technology, Kunming 650500, Yunnan, China; lpsun@kmust.edu.cn (L.S.); kgqml2012@163.com (Q.L.); baochangjun77@163.com (C.B.)

**Keywords:** wild edible mushrooms, free amino acids, nutrition, principal component analysis, Yunnan

## Abstract

Thirteen popular wild edible mushroom species in Yunnan Province, *Boletus bicolor*, *Boletus speciosus*, *Boletus sinicus*, *Boletus craspedius*, *Boletus griseus*, *Boletus ornatipes*, *Xerocomus*, *Suillus placidus*, *Boletinus pinetorus*, *Tricholoma terreum*, *Tricholomopsis lividipileata*, *Termitomyces microcarpus*, and *Amanita hemibapha*, were analyzed for their free amino acid compositions by online pre-column derivazation reversed phase high-performance liquid chromatography (RP-HPLC) analysis. Twenty free amino acids, aspartic acid, glutamic acid, serine, glycine, alanine, praline, cysteine, valine, methionine, phenylalanine, isoleucine, leucine, lysine, histidine, threonine, asparagines, glutamine, arginine, tyrosine, and tryptophan, were determined. The total free amino acid (TAA) contents ranged from 1462.6 mg/100 g in *B. craspedius* to 13,106.2 mg/100 g in *T. microcarpus*. The different species showed distinct free amino acid profiles. The ratio of total essential amino acids (EAA) to TAA was 0.13–0.41. All of the analyzed species showed high contents of hydrophobic amino acids, at 33%–54% of TAA. Alanine, cysteine, glutamine, and glutamic acid were among the most abundant amino acids present in all species. The results showed that the analyzed mushrooms possessed significant free amino acid contents, which may be important compounds contributing to the typical mushroom taste, nutritional value, and potent antioxidant properties of these wild edible mushrooms. Furthermore, the principal component analysis (PCA) showed that the accumulative variance contribution rate of the first four principal components reached 94.39%. Cluster analysis revealed EAA composition and content might be an important parameter to separate the mushroom species, and *T. microcarpus* and *A. hemibapha* showed remarkable EAA content among the 13 species.

## 1. Introduction

Mushrooms have long been favored as highly tasty, nutritive, and health-promoting foods [1,2,3]. While preferred to cultivated fungi, wild growing mushrooms are collected and consumed as a delicacy worldwide for their specific aroma and texture [4]. They are also an attractive source of food flavoring materials in soups and sauces due to their umami or palatable taste [5]. Moreover, a vast body of evidence indicates that wild edible mushrooms contain many biologically active compounds disclosing antioxidant, antibacterial, hepatoprotective, antiradical, antihyperglycemic, antiangiogenic, and even anti-inflammatory, antitumor, antiallergic, antiatherogenic, and hematological properties [6,7,8].

Yunnan Province is located in southwestern China, with an area of 394,000 km^2^ and an elevation of 76.4–6740 m. The climate is mild and rainy in summer and autumn, providing ideal conditions for fungal growth. Therefore, Yunnan Province is a specific region abundant in wild mushrooms and over 880 species are identified as being edible, which accounts for 80% of the edible species identified in China and around 40% in the world [9]. According to statistical data from the Ministry of Commerce of the People’s Republic of China, in the year 2012, 8963 metric tons of wild edible mushrooms from Yunnan Province were exported to other countries, 50% of which were exported to European countries. The total export amount reached USD 0.105 billion [10]. It can be seen that the export of wild edible mushrooms has become an important part of the edible mushroom industry in Yunnan Province. Recently, with the rapid urbanization and industrialization of this area, studies have drawn attention to the evaluation of mineral element content in wild mushrooms from Yunnan Province for food safety [11]. Nevertheless, the reports on nutrients of wild edible mushrooms from this area are rare [6]. Our knowledge on the value of wild edible mushrooms from Yunnan Province is still limited, compared to that of central and eastern Europe.

Amino acid composition is a reliable indicator of the nutritional value of food. Free amino acids are the main constituents of functionally essential compounds that are found in mushrooms. The most typical mushroom taste can be given by the nonvolatile compounds, such as free amino acids and soluble sugars [12,13]. The aims of this work were to determine the free amino acid compositions and quantify the identified amino compounds of 13 commonly consumed and commercially popular wild edible mushrooms grown in Yunnan Province. 

## 2. Results and Discussion

The 13 wild edible mushroom species studied in this paper, such as *Suillus placidus* (Spl), *Boletinus pinetorus* (Bpi), *Tricholoma terreum* (Tte), *Tricholomopsis lividipileata* (Tli), *Termitomyces microcarpus* (Tmi), *Amanita hemibapha* (Ahe), are considered the most delicious mushrooms by indigenous peoples for soup or fried with excess oil and salt for long-term consumption. These mushroom species are difficult for storage and transportation due to their crisp and tender texture. Therefore, they are commercially popular for the local markets in Yunnan Province. On the other hand, *Boletus bicolor* (Bbi), *Boletus speciosus* (Bsp), *Boletus sinicus* (Bsi), *Boletus craspedius* (Bcr), *Boletus griseus* (Bgr), *Boletus ornatipes* (Bor), and *Xerocomus* (Xer) are very important economic species for domestic and foreign trade, due to their bruise resistance and longer shelf life.

**Figure 1:**
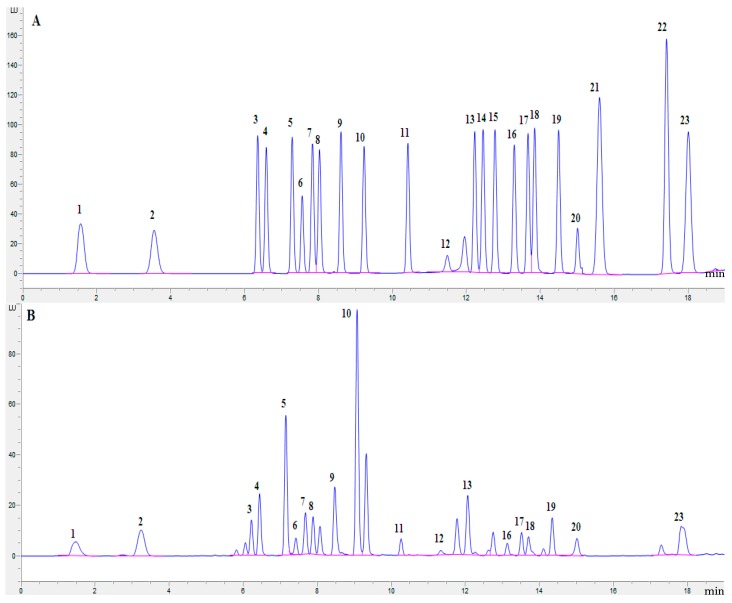
Typical chromatograms of free amino acid analysis by online pre-column derivazation RP-HPLC. (**A**) standard of 23 amino acids; (**B**) *Suillus placidus*. 1. aspartic acid; 2. glutamic acid; 3. asparagines; 4. serine; 5. glutamine; 6. histidine; 7. glycine; 8. threonine; 9. arginine; 10.alanine; 11. tyrosine; 12. cysteine; 13. valine; 14. methionine; 15. norvaline; 16. tryptophan; 17. phenylalanine; 18. isoleucine; 19. leucine; 20. lysine; 21. hydroxyproline; 22. sarcosine; 23. proline.

As shown in Table 1 and Figure 1, in almost all of the species it was possible to determine 20 free amino acids: alanine, arginine, aspartic acid, cysteine, glutamic acid, glycine, histidine, isoleucine, leucine, lysine, methionine, phenylalanine, proline, serine, threonine, tryptophan, tyrosine, valine, asparagine, and glutamine. The total free amino acid (TAA) contents in the analyzed samples ranged from 1462.6 mg/100 g in *B. craspedius* to 13,106.2 mg/100 g in *T. microcarpus* (Table 1). The average total free amino acid concentration of the 13 species was ca. 4345 mg/100 g. As far as we know, this is the first work revealing the presence of 20 essential and nonessential free amino acids in the referred wild edible mushroom species, which is very important considering their nutritional value, typical mushroom taste, and biological properties. In previous reports considering other edible mushroom species, the contents of free amino acids were considered to be less, only about 1000 mg/100 g of dry matter [4]. Leόn-Guzmán et al. [14] reported that the total free amino acid range of four wild edible mushrooms from Querétaro, México was ca. 2317–4741 mg/100 g. Ribeiro et al. [15] reported that the total free amino acid contents in 11 wild edible mushrooms from northeastern Portugal ranged from 153.09 mg/100 g in *F. hepatia* to 2267.32 mg/100 g in *B. edulis*, whereas, data from the literature showed ca. 897 mg/100 g of total free amino acids in *B. edulis* [16]. Kıvrak et al. [17] determined free amino acid contents in *Calvatia gigantean* as ca. 199.6 mg/100 g. It could be noted that up to 16,843 mg/100 g of total free amino acids were determined in five cultivated edible mushrooms, and the average content was 12,079 mg/100 g [12]. Concerning the species described above, the differences between the results in this study and those in published reports are assumed to be caused by the diversity of extraction, derivatization, or quantification methods used in the different studies. Nevertheless, these studies suggested that, as demonstrated in our work, the free amino acid contents in mushrooms were considerably divergent between species. In addition, the different geographical origin, growth conditions, and harvesting times of the analyzed species cannot be excluded.

Essential amino acid (EAA) contents in the analyzed species varied between 154.3 mg/100 g in *B. craspedius* and 5232.5 mg/100 g in *T. microcarpus* (Table 1). Eight kinds of essential amino acids were detected in 10 mushroom species with the exception of *B. sinicus*, *B. craspedius*, and *S. placidus*. The ratio of EAA/TAA was 0.11–0.40. According to the report of a Joint FAO/WHO/UNU expert consultation, 83.5 mg/(kg·D) of essential amino acids is recommended for an adult. We considered 60 kg as the weight of an average consumer, in agreement with the EU Scientific Committee for Food Adult Weight parameter, an intake of mushrooms equal to 300 g of fresh weight, which contains 30 g of dry matter, would provide ca. 1% to 27% of the FAO daily recommended dose.

Mushrooms are appreciated for their highly intense and delicious taste [18]. Among amino acids, the taste amino acids were those contributing to the typical mushroom taste. The delicious taste of mushrooms is primarily due to the presence of flavor amino acids (FAA) and other small molecules [15]. As shown in Table 1, FAA contents in the analyzed species varied between 281.1 mg/100 g in *B. craspedius* and 1866.5 mg/100 g in *T. microcarpus*, with the ratio of FAA/TAA being 0.07–0.22. Other taste compounds appeared to give sweet, bitter, or less intense sensations. Ser, Gly, Ala, and Pro are known to have a sweet taste, and the sweet amino acid (SAA) contents in the analyzed mushrooms ranged from ca. 485.3 mg/100 g in *T. lividipileata* to 2586.1 mg/100 g in *T. microcarpus*, with the ratio of SAA/TAA being 0.17–0.35. SAA are taste-active amino acids in mushrooms [16]. Ribeiro et al. [15] also noted that SAA contributes to mushroom flavor, on a large scale. Therefore, FAA and SAA might be responsible for the natural taste of these 13 species. Further sensory evaluations could be conducted to relate the known differences in tastes between these 13 species and the FAA and SAA compositions, to explore the puzzling question as to which taste compounds exhibit the typical taste of edible mushrooms, such as soluble carbohydrates, purine-5’-nucleotides, organic acids, and free amino acids [5]. 

Amino acids also display antioxidant activities in mushrooms [19]. The antioxidant capacities of the different amino acids might be rather different. Some studies proved that the hydrophobic amino acids (HAA) play an important role in antioxidant activities. A high content of HAA could enhance their antioxidant activity [20]. As shown in Table 1, all of the analyzed species in this study showed high contents of HAA (33%–54% of the total amino acids). Therefore, these mushrooms species could be expected to provide potent antioxidant materials, especially *B. speciosus*, *B. ornatipes*, *Xerocomus*, *T. microcarpus*, and *A. hemibapha*, which contained the highest HAA contents.

**Figure 2:**
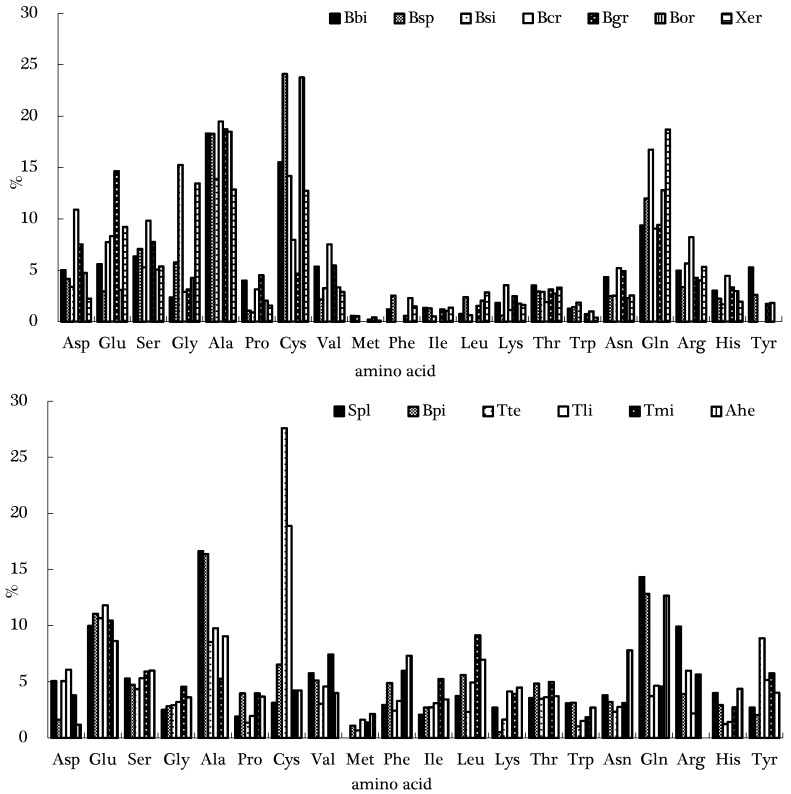
Amino acid profiles of the mushroom species. Abbreviations: Bbi, *B. bicolor*, Bsp, *B. speciosus*, Bsi, *B. Sinicus*, Bcr, *B. craspedius*, Bgr, *B. griseus*, Bor, *B. ornatipes*, Xer, *Xerocomus*, Spl, *S. placidus*, Bpi, *B. pinetorus*, Tte, *T. terreum*, Tli, *T. lividipileata*, Tmi, *T. microcarpus*, Ahe, *A. hemibapha*. Asp, aspartic acid, Glu, glutamic acid, Ser, serine, Gly, glycine, Ala, alanine, Pro, proline, Cys, cysteine, Val, valine, Met, methionine, Phe, phenylalanine, Ile, isoleucine, Leu, leucine, Lys, lysine, His, histidine, Thr, threonine, Asn, asparagines, Gln, glutamine, Arg, arginine, Tyr, tyrosine, Trp, tryptophan.

The quantification of the identified amino compounds is shown in Figure 2. It could be noted that all of the analyzed mushrooms revealed distinct quantitative profiles. Alanine, cysteine, glutamine, and glutamic acid were among the most abundant amino acids present in all species. Alanine was the main compound in *B. bicolor* (18.3%), *B. craspedius* (19.5%), *B. griseus* (18.7%), *S. placidus* (16.6%), and *B. pinetorus* (16.4%). Cysteine also seemed to have great importance, and it was the most abundant compound in *B. speciosus* (24.1%), *B. ornatipes* (23.8%), *T. terreum* (27.6%), and *T. lividipileata* (18.9%). Glutamine was the principal component in *B. Sinicus* (16.7%), *Xerocomus* (18.7%), and *A. hemibapha* (12.7%). Glutamic acid was the principal amino acid for *T. microcarpus* (10.4% of the total compounds). The amino acid profiles found in the literature for wild edible mushrooms were very distinct. However, it seemed that alanine, glutamine, and glutamic acid were often the major compounds. Our study is consistent with the previous works. However, as far as we know, no previous works showed cysteine as the principal compound in mushrooms.

Principal component analysis (PCA) is an unsupervised learning method that reduces the dimensionality of a data set by extracting only the most important information for analysis [21]. The accumulative variance contribution rate of the first four principal components was 94.39%, which reflected most of the information regarding the free amino acid compositional variability in the 13 wild edible mushrooms. In this study, only component loadings >0.75 were taken for interpretation [22]. The component loads showed that 70.81% of the variance was explained by PC1 (Table 2) and was mostly attributable to essential amino acids, except Thr, while additional amino acids, such as Ser, Pro, Asn, His, and Tyr, also displayed a contribution to this component. The PC2 accounted for13.16% of the total variance and positively loaded on Asp and Arg, showing 0.778 and 0.832 of the loading values, respectively. The percentages of variance explained by PC3 and PC4 were 6.31% and 4.11%, respectively. The loads on Gly and Gln were 0.880 and 0.945 in PC3, while, in Cys it was 0.883 in PC4.

**Figure 3:**
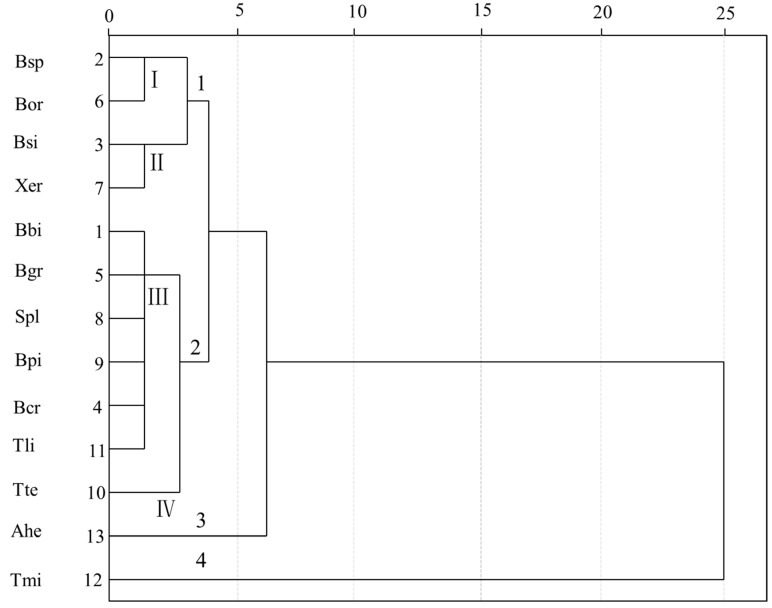
Tree diagram of the cluster analysis for the 13 mushroom species.

Cluster analysis undertaken with all the analytical parameters revealed four different clusters (Figure 3). Cluster 1 and 2 included four and seven mushroom species, respectively, while, cluster 3 and 4 contained only one species each, Ahe and Tmi, respectively. As shown in Table 2 and Figure 3, EAA played an important role in the clusters of the 13 mushroom species. Tmi in cluster 4 showed the highest content of EAA among the 13 mushrooms, and Ahe in cluster 3 was significantly higher than in the others. Cluster 1 was formed of four species and the range of EAA contents was 4234.3–5650.1 mg/100 g. Cluster 1 was further divided into two sub-clusters which were noted I and II. The EEA contents of Bsp and Bor in sub-cluster I showed no significant difference, while Bsi was similar to Xer in sub-cluster II. Cluster 2 produced two sub-clusters, III and IV, which included six and one mushroom, respectively. Tte in sub-cluster IV was different from the other six mushroom species in sub-cluster III, due to the high content of Tyr and low content of Gln in the Tte sample, except for the difference in EAA content. This was in accordance with the PCA analysis (Table 2).

## 3. Materials and Methods

### 3.1. Sampling

Samples of wild edible mushroom species were either collected from the field or purchased from the indigenous people who collect edible forest resources in the region or from the local markets in Yunnan Province during July and August 2014. Six matured specimens in good condition per species were collected. After collection, the voucher specimens were immediately transferred to the laboratory, usually within 12 h. Taxonomic identification followed that previously described by several authors [23,24]. Then, the fresh fruiting bodies were cleaned of forest debris using a plastic knife and were washed successively by running water and distilled water. The cleaned samples were frozen at −80 °C for 24 h, freeze dried, and grounded into fine powder (40 mesh). The milled samples were stored under vacuum in a brown desiccator until determination.

### 3.2. Determination of Free Amino Acid Composition

The free amino acid compositions of samples were determined by online pre-column derivazation RP-HPLC (Agilent 1260 series, Agilent Technologies, Santa Clara, CA, USA), recommended by Agilent Technologies (www.agilent.com/chem). The HPLC system was equipped with an Agilent G1315A FLD detector and a ZORBAX Eclipse-AAA column (3.0 mm × 150 mm, 3.5 μm, Agilent Technologies). The mobile phase was A: 40 mM NaH_2_PO_4_ (Ph 7.8) and B: CAN:MeOH:H_2_O (45:45:10). The mobile phase gradient and injector program are shown in Table 3 and Table 4. An FLD detector was set at 340 nm excitation, 450 nm emission, with PTM gain 10, and changed the signal to 266 nm excitation, 305 nm emission, with PTM gain 9 at 15.1 min. Amino acid identification was made by comparing the relative retention times of the peaks from samples with standards (standard of 23 amino acids, alanine, arginine, aspartic acid, cysteine, glutamic acid, glycine, histidine, isoleucine, leucine, lysine, methionine, phenylalanine, proline, hydroxyproline, serine, threonine, tryptophan, tyrosine, valine, norvaline, asparagine, glutamine, and sarcosine, from Agilent amino acid analysis kits). The results were expressed in mg/100 g dry weight (DW), calculated by the linear equations of each amino acid (*r*^2^ > 0.99, data not shown). 

Mushroom powder (0.1 g) samples were extracted by 20 mL of 75% (*v*/*v*) ethanol at 80 °C for 15 min. The resulting suspensions were centrifuged at 5000 rpm for 10 min. The supernatants were concentrated at 55 °C under reduced pressure and freeze-dried successively. The solid residues were dissolved by 15 mL 3% trichloroacetic acid solution with ultrasonication for 15 min. The solutions were centrifuged at 6000 rpm for 10 min and the supernatants were filtered through membrane filters with an average pore diameter of 0.22 μm for free amino acid composition determination by RP-HPLC.

### 3.3. Data Analysis

Determinations were performed in triplicate. Results were expressed as mean (standard deviation). Principal component analysis (PCA) and cluster analysis was conducted to interpret the obtained results of the 20 free amino acids in the 13 wild edible species by SPSS software (version 19.0, IBM, Chicago, IL, USA).

## 4. Conclusions

The aim of this work was to provide the free amino acid compositions and to achieve knowledge about the amino acid profiles of *B. bicolor*, *B. speciosus*, *B. sinicus*, *B. craspedius*, *B. griseus*, *B. ornatipes*, *Xerocomus*, *S. placidus*, *B. pinetorus*, *T. terreum*, *T. lividipileata*, *T. microcarpus*, *A. hemibapha* for the first time. All 13 analyzed mushroom species contained significant free amino acid contents, moderate essential amino acid contents, and high contents of hydrophobic amino acids, which may be important compounds contributing to the typical mushroom taste, nutritional value, and potent antioxidant properties of these wild edible mushrooms. Essential amino acid composition and content might be an important parameter to separate the species according to the PCA and cluster analysis, and *T. microcarpus* and *A. hemibapha* showed remarkable essential amino acid contents among the 13 species.

## Figures and Tables

**Table 1:** Concentrations (Milligrams per 100 g) of Free Amino Acids in Mushroom Species.

Species	Bbi	Bsp	Bsi	Bcr	Bgr	Bor	Xer	Spl	Bpi	Tte	Tli	Tmi	Ahe
Asp	169.6 (6.9)	186.7 (3.9)	164.1 (0.8)	159.3 (5.3)	232.5 (3.9)	200.7 (1.6)	126.7 (2.3)	113.1 (8.7)	42.1 (2.7)	182.1 (3.3)	145.8 (8.6)	497.3 (9.6)	63.7 (3.8)
Glu	189.7 (7.4)	132.5 (4.0)	374.7 (9.8)	121.8 (4.0)	452.8 (1.6)	131.3 (3.0)	520.5 (5.4)	222.3 (14.8)	285.6 (10.7)	382.5 (8.8)	283.0 (13.4)	1369.2 (16.0)	462.6 (10.9)
Ser	215.3 (6.2)	318.0 (5.5)	256.0 (9.5)	143.6 (5.5)	239.7 (0.5)	214.4 (3.5)	303.7 (3.0)	118.1 (8.8)	122.4 (5.3)	156.4 (4.4)	127.7 (7.0)	775.7 (13.4)	322.4 (22.1)
Gly	80.2 (2.6)	259.4 (1.7)	738.2 (30.2)	42.3 (1.9)	97.3 (0.5)	180.5 (4.1)	760.0 (4.8)	55.9 (4.3)	73.1 (3.4)	104.9 (1.7)	76.9 (3.1)	597.9 (10.4)	194.3 (5.7)
Ala	620.1 (15.6)	820.8 (7.9)	672.4 (7.9)	284.8 (9.2)	578.4 (2.3)	782.7 (6.3)	727.0 (5.8)	370.9 (27.6)	423.0 (18.6)	306.3 (6.0)	233.9 (10.1)	690.9 (10.2)	485.4 (13.5)
Pro	135.2 (6.5)	48.1 (2.8)	43.4 (7.0)	46.0 (1.3)	139.8 (7.7)	86.7 (0.7)	88.3 (1.5)	42.4 (1.3)	102.9 (2.8)	48.8 (4.1)	46.8 (2.7)	521.6 (43.4)	198.1 (20.5)
Cys	525.4 (17.8)	1082.4 (53.8)	686.4 (3.4)	116.2 (0.1)	144.7 (5.8)	1006.2 (45.7)	719.8 (26.7)	69.8 (4.8)	168.9 (7.6)	990.5 (5.7)	452.5 (43.6)	555.5 (36.7)	227.2 (32.0)
Val	181.3 (9.9)	97.2 (2.0)	157.4 (5.5)	109.8 (5.2)	169.0 (0.8)	141.5 (1.4)	163.3 (1.4)	128.6 (8.9)	132.4 (6.1)	109.3 (1.7)	109.8 (4.5)	971.8 (18.1)	214.4 (5.1)
Met	19.1 (0.6)	24.7 (0.1)	nd	nd	6.2 (0.2)	18.2 (0.9)	6.0 (0.1)	nd	28.2 (1.7)	24.0 (0.0)	38.6 (2.1)	180.4 (7.3)	114.7 (2.5)
Phe	40.8 (2.1)	113.9 (0.5)	nd	nd	17.2 (0.3)	97.3 (1.3)	83.7 (1.7)	65.5 (4.6)	126.6 (5.8)	87.0 (0.0)	79.1 (3.4)	785.8 (14.4)	392.5 (13.5)
Ile	45.8 (2.4)	58.3 (1.0)	25.0 (0.7)	nd	36.7 (0.0)	44.4 (0.4)	76.8 (1.5)	46.3 (2.9)	70.1 (3.8)	98.0 (2.0)	74.3 (3.4)	689.6 (10.2)	184.1 (4.6)
Leu	25.4 (1.5)	106.8 (1.2)	30.8 (0.3)	nd	47.3 (0.4)	86.3 (2.6)	160.5 (4.3)	83.4 (5.6)	145.0 (7.2)	83.3 (1.3)	118.3 (7.2)	1197.3 (6.1)	373.0 (12.8)
Lys	61.8 (2.3)	27.0 (3.0)	171.7 (0.3)	16.5 (0.0)	76.7 (2.6)	74.4 (21.1)	92.1 (4.6)	60.5 (8.8)	13.2 (3.3)	58.8 (1.1)	99.4 (9.7)	512.1 (10.4)	241.2 (14.9)
Thr	119.1 (3.8)	132.3 (2.2)	140.5 (3.0)	28.0 (1.1)	97.0 (0.1)	116.0 (2.4)	187.2 (16.1)	79.6 (5.7)	125.3 (5.8)	125.3 (3.0)	86.9 (3.8)	653.6 (9.1)	199.9 (4.6)
Trp	43.2 (3.1)	63.9 (0.2)	90.1 (8.3)	nd	23.0 (0.7)	42.1 (1.0)	22.4 (0.4)	69.1 (3.0)	81.3 (5.6)	37.0 (1.7)	36.3 (2.5)	241.9 (5.6)	145.6 (3.0)
Asn	146.4 (5.5)	111.5 (2.0)	122.3 (2.9)	76.2 (3.4)	152.0 (2.3)	97.3 (2.1)	143.9 (1.1)	85.0 (5.4)	82.7 (4.5)	83.4 (5.4)	66.0 (4.1)	408.0 (8.2)	417.7 (24.0)
Gln	316.8 (10.0)	538.2 (15.4)	810.3 (4.8)	132.4 (6.3)	291.3 (0.3)	541.4 (13.5)	1056.5 (1.2)	319.4 (0.2)	331.7 (16.9)	133.9 (2.2)	111.2 (7.5)	601.7 (15.3)	679.6 (15.4)
Arg	168.2 (9.1)	151.0 (3.2)	274.3 (10.8)	120.4 (4.3)	131.9 (1.1)	170.0 (18.6)	301.8 (9.4)	221.0 (14.9)	101.3 (2.9)	215.0 (9.3)	52.0 (3.6)	742.0 (20.6)	nd
His	102.3 (3.1)	100.6 (0.3)	81.6 (4.0)	65.3 (2.7)	103.1 (3.8)	125.9 (2.5)	109.9 (0.4)	89.3 (5.8)	75.8 (3.5)	44.6 (1.1)	34.1 (2.8)	357.5 (6.6)	234.8 (9.3)
Tyr	178.4 (8.7)	117.3 (1.2)	nd	nd	53.8 (0.1)	77.0 (2.5)	nd	60.3 (6.7)	52.9 (3.0)	318.4 (6.2)	124.0 (6.2)	756.4 (20.4)	216.6 (6.3)
TAA	3384.1	4490.6	4839.2	1462.6	3090.4	4234.3	5650.1	2300.5	2584.5	3589.5	2396.6	13,106.2	5367.8
EAA	536.5	624.1	615.5	154.3	473.1	620.2	792	533	722.1	622.7	642.7	5232.5	1865.4
EAA/TAA	0.16	0.14	0.13	0.11	0.15	0.15	0.14	0.23	0.28	0.17	0.27	0.40	0.35
FAA	359.3	319.2	538.8	281.1	685.3	332.0	647.2	335.4	327.7	564.6	428.8	1866.5	526.3
FAA/TAA	0.11	0.07	0.11	0.19	0.22	0.08	0.12	0.15	0.13	0.16	0.18	0.14	0.10
SAA	1050.8	1446.3	1710.0	516.7	1055.2	1264.3	1879.0	587.3	721.4	616.4	485.3	2586.1	1200.2
SAA/TAA	0.31	0.32	0.35	0.35	0.34	0.30	0.33	0.26	0.28	0.17	0.20	0.20	0.22
HAA	1593.1	2352.2	1615.4	556.8	1139.3	2263.3	2025.4	806.9	1197.1	1747.2	1153.3	5592.9	2189.4
HAA/TAA	0.47	0.52	0.33	0.38	0.37	0.54	0.36	0.35	0.46	0.49	0.48	0.43	0.41

nd, not detected; Bbi, *B. bicolor*; Bsp, *B. speciosus*; Bsi, *B. sinicus*; Bcr, *B. craspedius*; Bgr, *B. griseus*; Bor, *B. ornatipes*; Xer, *Xerocomus*; Spl, *S. placidus*; Bpi, *B. pinetorus*; Tte, *T. terreum*; Tli, *T. lividipileata*; Tmi, *T. microcarpus*; Ahe, *A. hemibapha*; TAA, total amino acid; FAA, flavor amino acid, containing Glu and Asp; SAA, sweet amino acids, containing Ser, Gly, Ala, and Pro; HAA, hydrophobic amino acids, containing Ala, Pro, Cys, Val, Met, Phe, Ile, and Leu; EAA, essential amino acids, were calculated as the total content of Val, Met, Phe, Ile, Leu, Lys, Thr, and Trp.

**Table 2:** Factor loadings after Varimax normalized rotation.

Elements	PC1	PC2	PC3	PC4
Asp	0.474	**0.778**	−0.058	0.304
Glu	0.700	0.612	0.259	−0.100
Ser	**0.762**	0.482	0.312	0.240
Gly	0.109	0.402	**0.880**	0.152
Ala	0.172	0.045	0.568	0.705
Pro	**0.868**	0.439	0.081	0.007
Cys	−0.134	0.148	0.155	**0.883**
Val	**0.767**	0.622	0.111	0.019
Met	**0.958**	0.205	0.015	0.005
Phe	**0.932**	0.307	0.084	0.028
Ile	**0.831**	0.537	0.066	0.030
Leu	**0.856**	0.479	0.116	0.007
Lys	**0.816**	0.451	0.276	−0.041
Thr	0.594	0.585	0.466	0.066
Trp	**0.874**	0.261	0.222	−0.036
Asn	**0.93**	−0.012	0.273	−0.055
Gln	0.190	−0.052	**0.945**	0.222
Arg	0.405	**0.832**	0.277	0.137
His	**0.922**	0.215	0.244	0.079
Tyr	**0.868**	0.445	−0.034	0.093
Eigenvalues	14.16	2.632	1.262	0.821
Variance	70.81%	13.16%	6.31%	4.12%
Cumulative	70.81%	83.97%	90.27%	94.39%

Note: Values in bold correspond to component loadings >0.75.

**Table 3:** The mobile phase gradient for on-line pre-column derivazation RP-HPLC analysis of amino acid composition.

Time (min)	Flow Rate (mL/min)	Mobile Phase A (%)	Mobile Phase B (%)
0	1	100	0
1.9	1	100	0
18.1	1	43	57
18.6	1	0	100
22.3	1	0	100
23.2	1	100	0
26	1	100	0

**Table 4:** The injector program for on-line pre-column derivazation RP-HPLC analysis of amino acid composition.

1	Draw 2.5 μL borate buffer from vial 1	8	Draw 0.5 μL FMOC from vial 4
2	Draw 0.5 μL samples from sample vials	9	Mix 4 μL in air, max speed, 6 times
3	Mix 3 μL in air, max speed, 2 times	10	Draw 0 μL ACN from vial 6
4	Draw 0 μL water from vial 2	11	Draw 32 μL water from vial 5
5	Draw 0.5 μL OPA from vial 3	12	Mix 18 μL in air, max speed, 6 times
6	Mix 3.5 μL in air, max speed, 6 times	13	Inject
7	Draw 0 μL water from vial 2		
